# Case Report: Pancreatic toxoplasmosis: role of endoscopic ultrasound in diagnosis

**DOI:** 10.3389/fgstr.2026.1763923

**Published:** 2026-06-04

**Authors:** Cosmas Rinaldi A. Lesmana, Randy Adiwinata, Jennifer Simca, Caroline Tanadi, Kevin Tandarto, Stella Marleen, Daniel Ruslim, Joyce Bratanata

**Affiliations:** 1 Division of Hepatobiliary, Department of Internal Medicine, Dr. Cipto Mangunkusumo National General Hospital, Faculty of Medicine Universitas Indonesia, Jakarta, Indonesia; 2 Gastrointestinal Cancer Center, Mochtar Riady Comprehensive Cancer Centre (MRCCC) Siloam Hospital Semanggi, Jakarta, Indonesia; 3 School of Medicine and Health Sciences, Atma Jaya Catholic University of Indonesia, Jakarta, Indonesia; 4 Department of Anatomical Pathology, Faculty of Medicine, Universitas Pelita Harapan, Jakarta, Indonesia; 5 Department of Anatomical Pathology, Mochtar Riady Comprehensive Cancer Centre (MRCCC) Siloam Semanggi, Jakarta, Indonesia; 6 Department of Radiology, Mochtar Riady Comprehensive Cancer Centre (MRCCC) Siloam Hospital Semanggi, Jakarta, Indonesia; 7 Department of Radiology, Faculty of Medicine, Tarumanegara University, Jakarta, Indonesia; 8 Division of Tropical and Infectious Disease, Department of Internal Medicine, Mochtar Riady Comprehensive Cancer Centre (MRCCC) Siloam Hospital Semanggi, Jakarta, Indonesia

**Keywords:** case report, diagnosis, endosonography, pancreas, toxoplasmosis

## Abstract

Toxoplasmosis can present as systemic disease affecting many organs, especially in immunocompromised patients. Most cases of toxoplasmosis present as encephalitis, while extracerebral toxoplasmosis is rare, particularly within the gastrointestinal tract. Here, we report the case of a 48-year-old patient with toxoplasmosis presenting as pancreatic nodules, chronic pancreatitis and encephalitis. He was referred to our hospital for the evaluation of pancreatic lesions. The patient had been previously hospitalized due to hemiparesis and dizziness. Contrast-enhance brain magnetic resonance imaging (MRI) revealed a space-occupying lesion in the cerebellum with surrounding edema. Positron emission tomography (PET) scan demonstrated focal hypermetabolic sign in the head and body of pancreas, raising suspicion for primary pancreatic malignancy. Serum lipase was mildly elevated to less than twice the upper limit of normal. Meanwhile, amylase, Ca 19-9, and CEA levels were within normal limits. Endoscopic ultrasound (EUS) was subsequently performed and revealed multiple hypoechoic nodules in the pancreatic head and body. EUS-guided fine needle biopsy (FNB) using a 22-G acquire needle was carried out for further evaluation. Histopathological examination showed chronic inflammation of the pancreatic tissue without evidence of malignancy.

## 1 Introduction

Toxoplasmosis is a globally distributed zoonotic infection caused by *Toxoplasma gondii*, a protozoan parasite belonging to the class Conoidasida, order Eucoccidiorida, and family Sarcocystidae (1). Toxoplasmosis is typically acquired through ingestion of *T. gondii* oocytes from contaminated soil, water, or food, as well as through consumption of undercooked meat. Following infection, the parasite can disseminate systematically (2). According to the Centers for Disease Control and Prevention, over 40 million people are estimated to be infected with *T. gondii* in Indonesia (3). The global seroprevalence for *T. gondii* infection is approximately 25.7%, with the greatest contributors being Africa (61.4%), followed by Oceania (38.5%) and South America (31.2%). On the other hand, lower rates were observed in the United States/Canada (17.5%) and Asia (16.4%) (4).

In immunocompetent individuals, toxoplasmosis is typically asymptomatic or presents as a mild, self-limiting illness characterized by fever and lymphadenopathy. In immunocompromised individuals, particularly those with Human Immunodeficiency Virus (HIV), latent *T. gondii* may reactivate and result in focal or disseminated disease. The most common manifestation in this population is toxoplasmic encephalitis (TE), especially in those with CD4 count <100 cells/μL and who are not receiving prophylaxis (5, 6). Extracerebral manifestations—including pneumonitis, myocarditis, chorioretinitis, and pancreatitis—are far less common and often under-recognized (7).

Pancreatic toxoplasmosis is exceedingly rare, with only a limited number of cases reported in the literature, highlighting its rarity and diagnostic challenge. It can clinically and radiologically mimic neoplastic lesions of the pancreas, leading to potential misdiagnosis and delayed treatment. Definitive diagnosis typically requires histopathological or cytological confirmation through tissue sampling.

Herein, we present a rare case of pancreatic toxoplasmosis in a 48-year-old male patient. The patient was initially suspected to have a pancreatic malignancy. However, the diagnosis was ultimately established via endoscopic ultrasound (EUS)-guided fine needle biopsy (FNB), which identified *T. gondii* organisms. This case emphasizes the importance of considering infectious etiologies, such as toxoplasmosis, in the differential diagnosis of pancreatic masses.

## 2 Case report

A 48-year-old male patient was referred to our hospital for evaluation of pancreatic nodules. He had been hospitalized at another institution with a three-week history of progressively worsening headaches, right-sided hemiparesis, dizziness, cognitive decline, and intermittent visual disturbances. Two days prior to admission, he experienced episodes of transient loss of consciousness. His medical history was significant for insulin-dependent Type diabetes mellitus. On presentation, the patient exhibited progressive neurological symptoms, including altered mentation and focal neurological deficits. He was afebrile and hemodynamically stable. Laboratory investigations showed normal serum amylase, CA 19-9, and CEA levels. Serum lipase levels was mildly elevated to approximately two times the upper limit of normal. No other significant laboratory abnormalities were noted. Contrast-enhanced magnetic resonance imaging (MRI) of the brain performed at the previous hospital demonstrated multiple ring-enhancing lesions, notably involving the left middle cerebellar peduncle and periventricular region of the left lateral ventricle. The lesion in the cerebellar peduncle measured up to 2.5 cm and was associated with significant perifocal edema, resulting in mass effect on the fourth ventricle and subsequent dilatation of the third and bilateral lateral ventricles, consistent with non-communicating hydrocephalus (**Figure 1**). The radiologic differential diagnoses included cerebral toxoplasmosis, pyogenic abscess, or metastatic disease.

**Figure 1:**
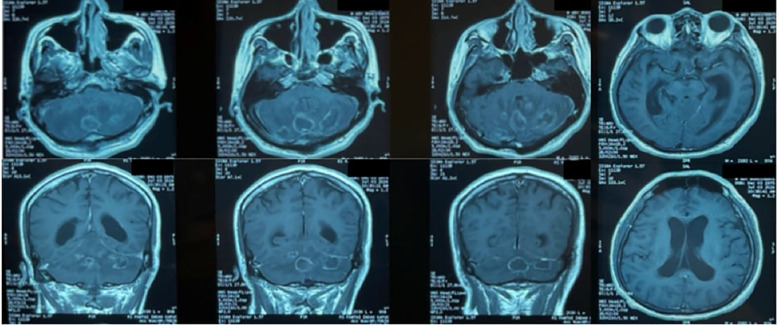
Patient’s brain MRI with contrast revealing multiple ring-enhancing lesions in the cerebellar peduncle and perivascular region of the left lateral ventricle Lesion in the cerebellar peduncle exerted mass effect on the fourth ventricle, causing dilatation of the third and bilateral lateral ventricles leading to the formation of a non-communicating hydrocephalus.

Based on the MRI findings, a positron emission tomography (PET) scan was conducted in our hospital, revealing a hypermetabolic lesion in the pancreatic head with additional focal uptake in the pancreatic body. There were no evidence of pancreatic duct dilation, fat stranding, peripancreatic or lymphadenopathy, and no radiologic features strongly suggestive of primary pancreatic malignancy were identified. In contrast, several of the previously noted ring-enhancing brain lesions demonstrated relative hypometabolism on PET imaging.

Several differential diagnoses were considered at this stage, including pancreatic adenocarcinoma, primary pancreatic lymphoma, infectious etiologies, and metastatic disease. Pancreatic adenocarcinoma was initially suspected due to the presence of a focal hypermetabolic lesion in the pancreatic head lesion on PET imaging. However, this diagnosis became less likely given the absence of characteristic radiology features, including pancreatic duct dilatation or vascular invasion, as well as normal tumor marker levels and only mildly elevated lipase.

Primary pancreatic lymphoma was also considered, particularly in light of the systemic and neurological manifestations. Nevertheless, imaging did not demonstrate typical features such as bulky homogeneous enlargement or significant lymphadenopathy, making this diagnosis less probable. Metastatic disease was another consideration but PET scan results failed to reveal a clear primary malignancy.

Infectious etiologies were therefore increasingly favored. In particular, as toxoplasmosis was considered the most likely diagnosis given the presence of multiple ring-enhancing brain lesions on MRI.

A key diagnostic challenge was the discordance between hypermetabolic pancreatic lesions and hypometabolic brain lesions of PET imaging, which complicated differentiation between malignant and infectious etiologies. Additionally, the absence of fever and largely unremarkable laboratory findings contributed to diagnostic uncertainty.

To further clarify the nature of the pancreatic lesion, the patient underwent EUS, which revealed a hypoechoic, irregular mass in the pancreatic head (**Figure 2**). EUS-guided FN was performed with a 22-gauge acquired needle (Boston Scientific). Three passes were made into the pancreatic head lesion under Doppler guidance to avoid vascular structures. Histopathological analysis revealed pancreatic tissue infiltrated by chronic inflammatory cells. Some cyst-like structures containing bradyzoites were found, which are pathognomonic for Toxoplasma gondii (**Figure 3**). Histopathological and serological evidence of Toxoplasma confirmed the diagnosis of pancreatic toxoplasmosis. **Figure 4** has summarized the timeline of the events for this case.

**Figure 2:**
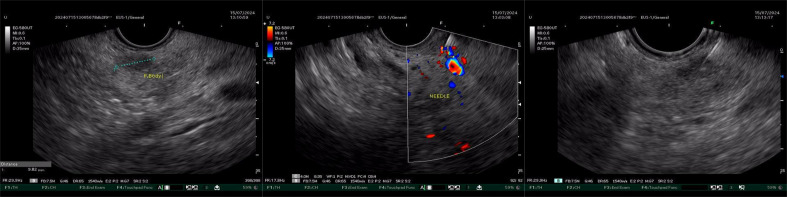
Endoscopic ultrasound results of the pancreas. The parenchyma appears inhomogeneous. Hypoechoic nodules are seen in the head and body regions. No dilatation of the pancreatic duct is observed. Currently, no clear tumor mass is visible. A biopsy (FNB) with a 22G needle was performed on the hypoechoic nodules (head and body).

**Figure 3:**
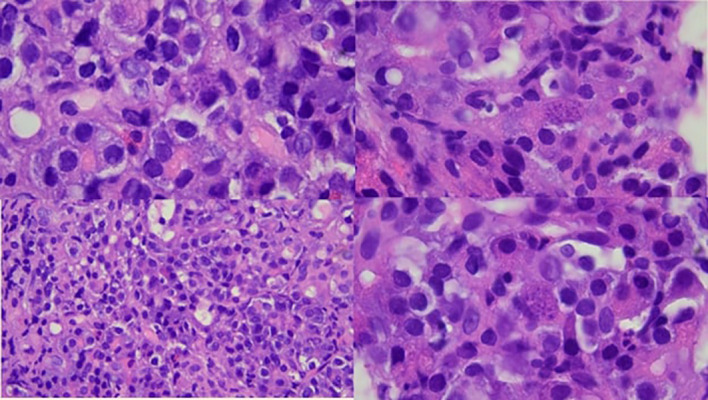
The pancreatic biopsy specimen shows fragments of pancreatic tissue that are partially infiltrated by chronic inflammatory cells. There are also areas that present a notable image of a cyst containing bradyzoites.

**Figure 4:**
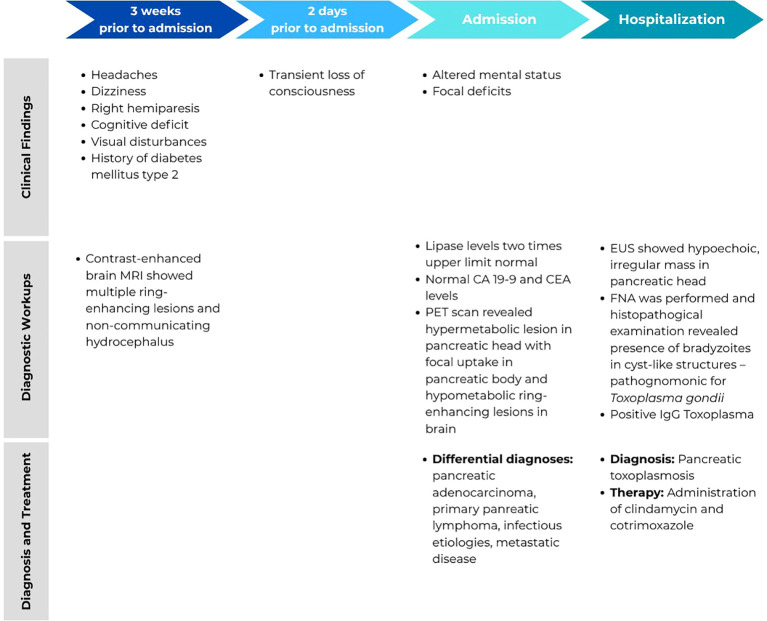
Clinical case timeline.

The patient was subsequently initiated on standard anti-toxoplasmosis therapy, including clindamycin 3x600 mg and cotrimoxazole 3x1920 mg, along with intravenous antibiotics (meropenem 3x1 gram and moxifloxacin 1x400 mg), antifungal therapy (fluconazole 1x200 mg IV drip), and supportive care. During hospitalization, the patient’s condition deteriorated due to sepsis in the setting of multiple concurrent infections, including cytomegalovirus infection, suspected tuberculosis, and pneumocystis pneumonia. The clinical course was further complicated by multiorgan failure and the development of acute respiratory distress syndrome.

## 3 Discussion

*Toxoplasma gondii* is an opportunistic protozoan capable of affecting nearly any organ system in immunocompromised individuals, with the central nervous system being the most commonly involved site. In contrast, extracerebral manifestations—particularly those involving the gastrointestinal tract—are uncommon (7).

Pancreatic involvement by *T. gondii* is exceedingly rare and typically presents as pancreatitis, mass-like lesions, or nodules that may mimic neoplastic processes. To date, no epidemiological studies have specifically reported the incidence of pancreatic toxoplasmosis; available knowledge is limited to isolated case reports. Ahuja et al. described a case of necrotizing pancreatitis associated with disseminated toxoplasmosis in a 34-year-old AIDS patient who developed multiorgan failure (8). Other reports have documented pancreatitis secondary to toxoplasmosis in patients undergoing immunosuppressive therapy or solid organ transplantation. In such cases, *T. gondii* may directly invade pancreatic tissue, triggering inflammation and necrosis. Notably, in many of these cases, the diagnosis was established only postmortem (9, 10). In HIV-infected individuals, reactivation of latent toxoplasmosis is the likely pathophysiological mechanism, while in transplant recipients, primary infection with *T. gondii* from donor tissue may occur. The pancreas, particularly its endocrine β-cells, may be targeted directly by the parasite or indirectly through immune-mediated mechanisms (10). In our case, we could not confirm the immunodeficiency status of the patient since the patient rejected tests for HIV; which became the major limitation of our case report and for further elaborated management.

Pancreatic toxoplasmosis can manifest as nodular lesions that are often radiologically indistinguishable from malignant neoplasms. Imaging modalities such as computed tomography (CT), and MRI are commonly used in the initial evaluation,with PET serving as an adjunct in selected cases. Among these modalities, MRI offers superior sensitivity (96.9%) and specificity (90.8%) compared to CT (sensitivity 80.6%, specificity 86.4%) for characterizing pancreatic lesions (11).

Contrast-enhanced CT remains widely accessible and is often the first-line imaging tool, with a reported sensitivity of 80.6% and specificity of 86.4% for the detection of pancreatic tumors. MRI offers superior soft tissue contrast and has demonstrated higher diagnostic accuracy, with a sensitivity of 96.9% and specificity of 90.8% for characterizing pancreatic lesions (11). PET using 18F-fluorodeoxyglucose (FDG) is valuable in identifying metabolically active lesions and assessing for systemic involvement. It is particularly useful when conventional imaging is inconclusive or when multiple lesions are present. However, PET has limited specificity, as inflammatory and infectious lesions—including toxoplasmosis—may also exhibit increased FDG uptake, thereby mimicking malignancy. The reported sensitivity and specificity of FDG-PET for detecting pancreatic cancer range from 85% to 94% and 65% to 97%, respectively, depending on the study population and lesion type. In the context of pancreatic toxoplasmosis, PET may yield false-positives due to the inflammatory response associated with parasitic infection (12).

EUS has emerged as a cornerstone modality in the evaluation of pancreatic lesions, especially when conventional imaging such as CT or MRI yields inconclusive results. By combining high-resolution imaging with real-time tissue sampling, EUS offers both diagnostic and therapeutic potential. EUS may detect small pancreatic lesions <2–3 cm in diameter (13). According to multiple studies, the diagnostic performance of EUS varies with lesion size. For pancreatic nodules measuring between 10 and 20 mm, sensitivity ranges from approximately 76% to 92%, with specificity reported as high as 93.8% to 100%. In contrast, for nodules 10 mm or smaller, sensitivity ranges from 40% to 100%, while specificity remains relatively high, between 80% and 100% (14). When EUS is combined with fine-needle aspiration (FNA) or fine-needle biopsy (FNB), its diagnostic accuracy is significantly enhanced. A recent meta-analysis reported that EUS-FNA achieves a sensitivity of 94.8% and specificity of 98.6% in diagnosing pancreatic cancer (15). EUS-FNB, which allows for the collection of core tissue samples, has shown comparable diagnostic accuracy, with pooled sensitivity and specificity rates of 90.8% and 96.5%, respectively. These techniques are particularly valuable in distinguishing malignant from benign lesions, guiding clinical management decisions. Despite its advantages, EUS also has limitations. Its diagnostic accuracy can be operator-dependent, and certain lesion characteristics, such as small size (<20 mm) or ill-defined margins, may reduce the effectiveness of EUS-guided tissue acquisition. In such cases, the use of contrast agents and the involvement of experienced operators can enhance diagnostic yield (16). In our case, the histopathological examination of the tissue was consistent with pancreatic toxoplasmosis. Polymerase chain reaction (PCR) testing for *Toxoplasma* was not performed, as the histopathological findings were considered sufficient for diagnosis. However, the absence of molecular confirmation represents a limitation in our case.

The treatment of pancreatic toxoplasmosis follows the principles of systemic toxoplasmosis management. The standard regimen consists of pyrimethamine (200 mg on day 1, followed by 75 mg/day if weight ≥60 kg or 50 mg/day if <60 kg) combined with sulfadiazine (3000 mg twice a day if weight ≥60 kg or 2000 mg twice a day of <60 kg) and folinic acid 10–15 mg/day for six weeks. Alternative regiments include pyrimethamine in combination with clindamycin (600–900 mg four times a day) and folinic acid or trimethoprim (5 mg/kg twice a day)/sulfamethoxazole (25 mg/kg twice a day). In cases of intolerance, atovaquone (1500 mg twice a day) in combination with pyrimethamine and folinic acid may be considered (17). In our case, the standard sulfadiazine–pyrimethamine regimen was not administered due to its unavailability in Indonesia. Additionally, atovaquone was also not accessible. Therefore, an alternative regimen consisting of clindamycin and cotrimoxazole was initiated.

## 4 Conclusion

Pancreatic toxoplasmosis is a rare manifestation of *Toxoplasma gondii* infection. Its clinical and radiologic presentation may closely mimic pancreatic neoplasia, posing a significant diagnostic challenge. This case highlights the critical role of EUS with FNB in the evaluation of pancreatic lesions, enabling definitive diagnosis through tissue sampling. Clinicians should maintain a high index of suspicion for infectious etiologies, including toxoplasmosis, in the differential diagnosis of pancreatic masses.

## Patient perspective

The patient was initially conscious and able to participate in clinical decision-making. However, the patient declined HIV testing during the early stage of hospitalization. As his condition progressed, he developed worsening neurological symptoms and was no longer able to provide further input. The patient’s family described the initial symptoms, particularly progressive neurological deficits and episodes of loss of consciousness, as distressing and alarming. They also reported that the period of diagnostic uncertainty, including concerns about possible malignancy, contributed to significant anxiety. Despite medical management, the patient’s rapid clinical deterioration and the development of multiple complications were emotionally overwhelming for the family.

## Data Availability

The original contributions presented in the study are included in the article/supplementary material. Further inquiries can be directed to the corresponding author.

## References

[1] Smith NC Goulart C Hayward JA Kupz A Miller CM van Dooren GG . Control of human toxoplasmosis. Int J Parasitol. (2021) 51:95–121. doi: 10.1016/j.ijpara.2020.11.001 33347832

[2] Tenter AM Heckeroth AR Weiss LM . Toxoplasma gondii: from animals to humans. Int J Parasitol. (2000) 30:1217–58. doi: 10.1016/s0020-7519(00)00124-7 11113252 PMC3109627

[3] Centers for Disease Control and Prevention . About toxoplasmosis (2024). Available online at: https://www.cdc.gov/toxoplasmosis/about/index.html (Accessed October 17, 2025).

[4] Molan A Nosaka K Hunter M Wang W . Global status of Toxoplasma gondii infection: systematic review and prevalence snapshots. Trop BioMed. (2019) 36:898–925. 33597463

[5] Dian S Ganiem AR Ekawardhani S . Cerebral toxoplasmosis in HIV-infected patients: a review. Pathog Glob Health. (2023) 117:14–23. doi: 10.1080/20477724.2022.2083977 35694771 PMC9848325

[6] Lau A Jain MK Chow JY Kitchell E Lazarte S Nijhawan A . Toxoplasmosis encephalitis: a cross-sectional analysis at a U.S. safety-net hospital in the late cART era. J Int Assoc Provid AIDS Care. (2021) 20:23259582211043863. doi: 10.1177/23259582211043863 34663116 PMC8529305

[7] Pastorello RG Costa A Sawamura MVY Nicodemo AC Duarte-Neto AN . Disseminated toxoplasmosis in a patient with advanced acquired immunodeficiency syndrome. Autops Case Rep. (2018) 8:e2018012. doi: 10.4322/acr.2018.012 29588907 PMC5861962

[8] Ahuja SK Ahuja SS Thelmo W Seymour A Phelps KR . Necrotizing pancreatitis and multisystem organ failure associated with toxoplasmosis in a patient with AIDS. Clin Infect Dis. (1993) 16:432–4. doi: 10.1093/clind/16.3.432 8452954

[9] Nasser QJ Power RE Eng MP Hickey DP Little DM . Toxoplasmosis after a simultaneous pancreas and kidney transplantation. Transplant Proc. (2004) 36:2843–4. doi: 10.1016/j.transproceed.2004.10.009 15621164

[10] Oz HS . Toxoplasmosis, pancreatitis, obesity and drug discovery. Pancreat Disord Ther. (2014) 4:138. 25530920 PMC4270089

[11] Expert Panel on Gastrointestinal Imaging Fábrega-Foster K Kamel IR Horowitz JM Arif-Tiwari H Bashir MR . ACR appropriateness criteria® Pancreatic cyst. J Am Coll Radiol. (2020) 17:S198–s206. doi: 10.1016/j.jacr.2020.01.021 32370963

[12] Jones MJ Buchanan AS Neal CP Dennison AR Metcalfe MS Garcea G . Imaging of indeterminate pancreatic cystic lesions: a systematic review. Pancreatology. (2013) 13:436–42. doi: 10.1016/j.pan.2013.05.007 23890144

[13] Sakamoto H Kitano M Kamata K El-Masry M Kudo M . Diagnosis of pancreatic tumors by endoscopic ultrasonography. World J Radiol. (2010) 2:122–34. doi: 10.4329/wjr.v2.i4.122 21160578 PMC2999320

[14] Yoshida T Yamashita Y Kitano M . Endoscopic ultrasound for early diagnosis of pancreatic cancer. Diagnostics (Basel). (2019) 9:81. doi: 10.3390/diagnostics9030081 31344904 PMC6787710

[15] Banafea O Mghanga FP Zhao J Zhao R Zhu L . Endoscopic ultrasonography with fine-needle aspiration for histological diagnosis of solid pancreatic masses: a meta-analysis of diagnostic accuracy studies. BMC Gastroenterol. (2016) 16:108. doi: 10.1186/s12876-016-0519-z 27580856 PMC5007683

[16] Yang Y Li L Qu C Liang S Zeng B Luo Z . Endoscopic ultrasound-guided fine needle core biopsy for the diagnosis of pancreatic Malignant lesions: a systematic review and meta-analysis. Sci Rep. (2016) 6:22978. doi: 10.1038/srep22978 26960914 PMC4785370

[17] Konstantinovic N Guegan H Stäjner T Belaz S Robert-Gangneux F . Treatment of toxoplasmosis: current options and future perspectives. Food Waterborne Parasitol. (2019) 15:e00036. doi: 10.1016/j.fawpar.2019.e00036 32095610 PMC7033996

